# Assessing the impact of feature communication in swarm perception for people re-identification

**DOI:** 10.3389/frobt.2025.1671952

**Published:** 2025-12-02

**Authors:** Miquel Kegeleirs, Ilyes Gharbi, Marios Kaplanis, Lorenzo Garattoni, Gianpiero Francesca, Mauro Birattari

**Affiliations:** 1 IRIDIA, Université libre de Bruxelles, Brussels, Belgium; 2 R&D, Toyota Motor Europe, Brussels, Belgium

**Keywords:** swarm robotics, swarm perception, distributed systems, robot communication, people re-id

## Abstract

Swarm perception enables a robot swarm to collectively sense and interpret the environment by integrating sensory inputs from individual robots. In this study, we explore its application to people re-identification, a critical task in multi-camera tracking scenarios. We propose a decentralized, feature-based perception method that allows robots to re-identify people across different viewpoints. Our approach combines detection, tracking, re-identification, and clustering algorithms, enhanced by a model trained to refine extracted features. Robots dynamically share and fuse data in a decentralized manner, ensuring that collected information remains up to date. Simulation results, measured by the cumulative matching characteristics (CMC) curve, mean average precision (mAP), and average cluster purity, show that decentralized communication significantly improves performance, enabling robots to outperform static cameras without communication and, in some cases, even centralized communication. Furthermore, the findings suggest a trade-off between the amount of data shared and the consistency of the Re-ID.

## 1 Introduction

Swarm perception refers to the ability of a robot swarm (Beni, 2005; Şahin, 2005; Dorigo et al., 2014) to leverage the sensory input of individual robots and achieve a collective understanding of the environment. Due to their distributed nature, robot swarms can collectively gather, share, and update information about their surroundings in a scalable, flexible, and fault-tolerant manner (Brambilla et al., 2013). This can be particularly advantageous in people (re)identification and tracking scenarios, especially in environments with unknown structure where static methods, which rely on strategic sensor placement or predefined path planning, become ineffective (Robin and Lacroix, 2016).

In the general case, tracking people across multiple cameras falls within the domain of multi-target, multi-camera tracking (MTMCT) (Amosa et al., 2023). Person re-identification (Re-ID) specifically addresses MTMCT problems in which people must be matched across multiple non-overlapping cameras (Tang et al., 2017; Ristani and Tomasi, 2018; Gaikwad and Karmakar, 2021). Traditional person re-identification (Re-ID) methods are designed for static CCTV cameras capturing low-resolution video feeds. These systems typically rely on whole-body feature extraction, making them sensitive to occlusions, lighting changes, pose variations, and clothing alterations (Ye et al., 2022). In contrast, robot swarms offer dynamic and adaptive perception. Their mobility and compact size allow them to reposition in response to crowd movement, capture scenes from closer and varied viewpoints, and scale easily by adding more units without requiring fixed infrastructure. Unlike static CCTV cameras, robots can also interact directly with people—providing guidance, assistance, or real-time information. However, swarm perception presents challenges, particularly in integrating and fusing observations collected along diverse and unsynchronized trajectories.

In this paper, we investigate how communication between robots impacts the performance of decentralized person re-identification (Re-ID) in robot swarms. We propose a feature-based decentralized perception framework that integrates detection, tracking, Re-ID, and clustering algorithms, enhanced by a model trained for robust visual feature extraction. Although our framework does not employ state-of-the-art models for detection, tracking, or re-identification, it is intentionally built upon well-established and reproducible components. This choice is motivated by the need to isolate and rigorously study the impact of inter-robot communication on collective perception in decentralized swarm systems. By controlling for the complexity and variability of individual modules, we ensure that observed effects can be attributed to communication mechanisms rather than fluctuations in module performance. This approach naturally limits absolute perception performance, yet provides a robust and interpretable basis for analyzing system-level coordination, adaptability, and resilience. Moreover, the modular structure of our framework allows any component to be replaced with a more advanced alternative, facilitating future studies that combine communication mechanisms with high-performance perception models.

To assess the role of communication, we systematically vary the communication range and analyze its impact on the ability to accurately re-identify people and on the consistency of clustering observations across the swarm. We conduct our evaluation in a simulated conference-like environment, which provides a dynamic and complex setting where Re-ID is particularly valuable. Furthermore, we compare the swarm’s performance to that of CCTV-based systems, analyzing both communication-enabled and isolated camera setups to underscore the advantages and limitations of decentralized robot swarms.

## 2 Related work

In swarm robotics, extensive research has been devoted to understanding collective behaviors (Trianni and Campo, 2015; Brambilla et al., 2013; Garattoni and Birattari, 2016) and collective decision making (Strobel et al., 2018; Valentini et al., 2016b), often highlighting the crucial role of perception. For example, studies by Valentini et al. (2016a) and Zakir et al. (2022) explore swarm perception in the context of collective decision making. Collective perception has received increasing attention, particularly in the context of (semi-)autonomous vehicles (Günther et al., 2015; Günther et al., 2016; Thandavarayan et al., 2019) and monitoring systems (Fiore et al., 2008; Choi and Savarese, 2012; Montero et al., 2023). In monitoring, person re-identification (Zheng et al., 2016) plays a crucial role, as collective perception requires agents to reach a consensus on the identity of detected and tracked people, enabling data fusion. Recent advances primarily leverage deep-learning techniques (Ye et al., 2022), especially feature-embedding methods such as those based on triplet loss and its variations (Hermans et al., 2017). Triplet loss has been a key framework for learning discriminative feature representations, ensuring that people remain distinguishable under challenging conditions such as occlusions, pose variations, and lighting changes (Cheng et al., 2016; Zeng et al., 2020). Although most re-identification research has focused on stationary cameras in surveillance systems (Ukita et al., 2016; Koide et al., 2017), mobile robots equipped with cameras offer the advantage of dynamic repositioning, enabling multi-view observations and closer interactions (Murata and Atsumi, 2018). Research has primarily explored single-robot applications, particularly in human-robot interaction and service robotics (Pinto et al., 2023; Carlsen et al., 2024). Face Re-ID improves the ability of a robot to interact personally with users (Wang et al., 2019), while recent work has integrated voice Re-ID to further improve recognition (Lu et al., 2024). Beyond recognition, robots can also follow users to provide continuous assistance (Ye et al., 2023). For instance, the CARPE-ID framework enables person re-identification despite occlusions and clothing changes, allowing a single robot to reliably track a person (Rollo et al., 2024). Preliminary studies have also explored multi-robot (Popovici et al., 2022) and swarm-based approaches (Kegeleirs et al., 2024a; b), highlighting promising directions for future advancements. However, most prior efforts emphasize the performance of individual models rather than their integration into robust and scalable systems. In contrast, our work focuses on the system-level effectiveness of decentralized swarm perception using readily available algorithms.

## 3 Methods

In the proposed method, robots acquire data and share it with their peers whenever they are within communication range.

### 3.1 Individual data acquisition

**FIGURE 1:**
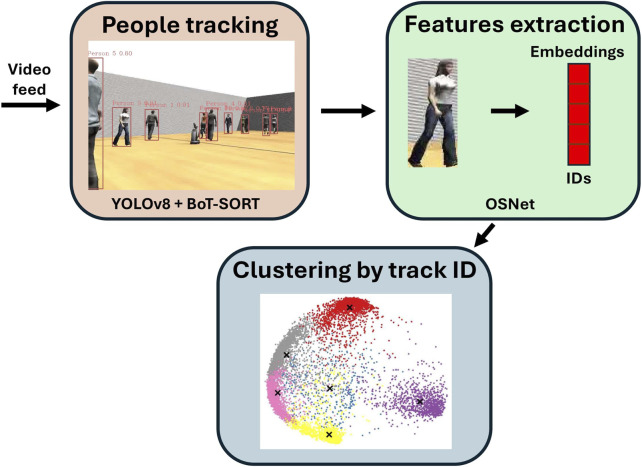
Overview of the individual data acquisition process.

Each robot independently performs re-identification and tracking using the video stream from its onboard camera. This process follows a three-step pipeline, as illustrated in Figure 1.

First, people are detected using YOLOv8 (Varghese and Sambath, 2024) trained on COCO (Lin et al., 2014). Second, the BoT-SORT tracking algorithm (Aharon et al., 2022) assigns a unique ID to each detected person and continues to track them as long as they remain within the field of view of the camera. Third, embeddings for each person are computed using the OSNet model (Zhou et al., 2021). As the pre-trained weights lack discriminative power, we fine-tuned the model on three datasets: Market1501 (Zheng et al., 2015), DukeMTMC-reID (Ristani et al., 2016; Gou et al., 2017), and CUHK03 (Li et al., 2014). This training enhances the discriminative power of the embeddings by ensuring that those corresponding to the same person are positioned closer together in the feature space, while those of different ones are farther apart. Embeddings are then clustered based on the IDs assigned by BoT-SORT. Clusters sharing the same ID are merged, so each resulting cluster ideally contains all detections and associated embeddings corresponding to a single person. When a new ID is to be assigned to a person, their embeddings are compared to all existing clusters using a distance-based merging algorithm: (i) the distance between the new embeddings and all existing clusters is computed—as the distance between their means. (ii) If the distance between the new embeddings and an existing cluster is below a predefined threshold (0.2), the new data are merged into this cluster, with the new ID being appended to the cluster’s known IDs. (iii) If no sufficiently similar cluster is found, a new cluster is created and initialized with the new ID. At any given time, the resulting set of clusters constitutes the robot’s people database.

### 3.2 Inter-robot data sharing

**FIGURE 2:**
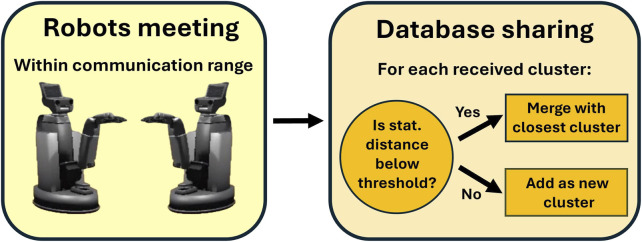
Overview of the inter-robot data sharing process.

When robots meet, they exchange their respective databases of people. The received clusters are integrated into the robot’s database using a distance-based merging algorithm similar to the one described above: (i) For each new cluster, the distance to all existing clusters is computed—as the distance between their means. (ii) If the distance between a new cluster and an existing one is below a predefined threshold, the two clusters are merged. This aims at grouping in a single cluster the embeddings that represent a same person, regardless of the robots that collected them. (iii) If the distance between a new cluster and each of the existing clusters exceeds the threshold, the new cluster is added as a distinct new person. Figure 2 summarizes this process.

### 3.3 Exploration behavior

Exploration behavior plays a crucial role in swarm-based perception, particularly in how data is collected and shared across the swarm. Robots can actively influence coverage and observation overlap by adjusting their movement patterns, potentially intensifying search in under-observed areas. However, in this specific study, to isolate and evaluate the direct impact of communication rates on decentralized person re-identification, we adopt a random walk exploration strategy. In particular, we selected ballistic motion, a straightforward implementation commonly used in previous studies (Francesca et al., 2014; 2015; Kegeleirs et al., 2019; Spaey et al., 2020). Random walk is a widely used baseline in swarm robotics due to its simplicity, decentralization, and unbiased spatial coverage (Dimidov et al., 2016; Schroeder et al., 2017). It provides a clean and neutral framework for analysis, avoiding confounding effects introduced by more complex or adaptive behaviors, and allowing us to attribute performance differences primarily to communication dynamics.

## 4 Experimental Setup

To evaluate the swarm’s ability to perform re-ID using the method presented in Section 3, we designed a series of simulated experiments in which Toyota HSR robots navigate within a closed environment resembling a conference venue, where multiple people move freely. In particular, we assess how communication enables the robot swarm to maintain a coherent and robust shared database of the tracked people.

### 4.1 Material

The environment and people are simulated in Unity, while robot movements are simulated in ARGoS3 (Pinciroli et al., 2012) and mirrored in Unity *via* ROS communication. The environment is a 625 m^2^ empty square room with bright lighting, enclosed by 3 m high walls. Additionally, we consider a second environment identical to the first but featuring five obstacles: 5 m × 3 m x 0.2 m panels that obstruct both movement and visibility. People move randomly at speeds ranging from 0.5 to 1.5 m/s, while robots execute a random walk with a linear speed of 0.3 m/s and an angular speed of about 0.2 rad/s, ensuring stable tracking performance during turns.

### 4.2 Protocol

For each environment, we consider two group sizes: 6, and 50 people. For each combination of environment and group size, we evaluate two swarm sizes: 4 and 8 robots, except in the 6-person scenario, where only 4 robots are used. This results in a total of 6 unique experiments. Additionally, four CCTV cameras are positioned at the room’s corners, facing the center, to capture video footage for comparison with the robots’ performance. For experiments with 50 people and 8 robots, four extra CCTV cameras are placed at the center of the room, each facing a different corner. Each experiment is a 5-min simulation during which robots and people move freely while avoiding obstacles. The robots continuously record video data from their onboard cameras, while CCTV cameras record from fixed positions. Robot positions are also logged solely to verify the communication range during video replay. After the simulation, videos produced by each robot are processed independently and sequentially in a synchronous way. Synchronization ensures that when robots exchange information, they refer to the same time window. By doing so, robots generate clusters of features as described in Section 3. To prevent excessive data exchange when robots remain in proximity for extended periods, communication occurs at most every 5 s. We assume error-free communication. In practice, limited packet loss should have minimal impact because robots transmit their full data payload at every exchange, allowing missing items to be recovered in subsequent exchanges. Exchanges are modeled as instantaneous relative to a simulation step (all data are synchronized before the next step), but they are limited to occurring no more frequently than once every 5 s, preventing unrealistically high data rates. We consider four communication settings: no communication and three communication ranges of 1.5 m, 5 m, and 10 m, with wider ranges associated with a higher number of data exchanges. CCTV cameras process their video feeds synchronously using the same algorithm as the robots, but only two communication settings are considered: no communication and centralized communication, where all cameras exchange data every 5 s.

For each experiment and communication setting, we compute the cumulative matching characteristics (CMC) curve, the mean average precision (mAP) and the average cluster purity for both robots and CCTV cameras, then aggregate the results accordingly. CMC@k evaluates top-k identification accuracy—how often the first correct identity appears within the top-k ranks. The mAP evaluates retrieval quality over the full ranking—how completely and how early all correct matches are returned. The CMC curve and mAP are computed using 10% of all images observed by the robots during the experiment as queries, with a minimum of 1,000 and a maximum of 5,000 queries. Cluster purity quantifies the extent to which each cluster contains only a single class. It is calculated as the proportion of the most represented ground-truth ID within each cluster. If duplicate IDs occur, only the largest cluster is considered. IDs that are not detected are penalized by assigning them a 0% proportion.

## 5 Experimental results

**FIGURE 3:**
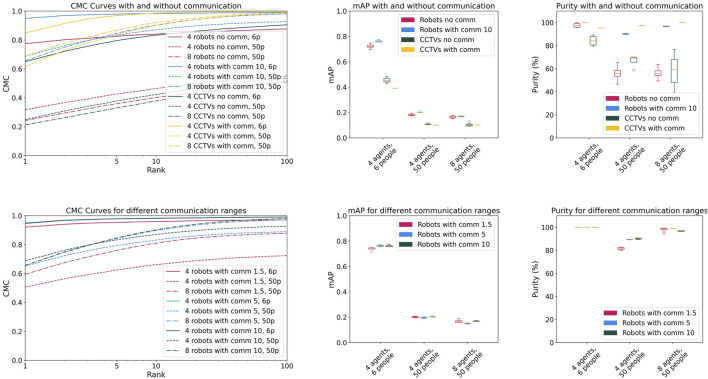
CMC, mAP, and purity (up) with and without communication and (down) for different communication ranges.

We first examine the impact of communication on the performance (see Figure 3).

Without communication, robots outperform CCTV cameras on both the CMC curve and in terms of purity/mAP. This difference is particularly pronounced in experiments with 6 people. This is particularly noteworthy given that both systems rely on the same lightweight and standard algorithmic components—highlighting the practical benefits of mobility and varied viewpoints available to decentralized robotic agents. Although CCTV cameras have a wider field of view, which allows them to observe more people simultaneously, frequent occlusions reduce the consistency of their clusters. Overall, results without communication remain relatively low in scenarios with 50 people. However, introducing communication greatly improves the robots’ performance, particularly in the 50-person experiments. This demonstrates that by exchanging information, robots enhance cluster quality—both by detecting new people and by refining previously detected persons through correct clustering of the data received from their peers. Centralized communication enables CCTV cameras to achieve slightly better results in the 50-people experiments, but they are outperformed by the robots in the 6-people experiment. These results suggest that, although centralized communication lets CCTV cameras collect more information, the extra data does not drastically improve clustering quality. In particular, their broader field of view helps when 50 people are present—enabling them to monitor many individuals at once—but this benefit largely vanishes in the 6-person experiments.

Also, cameras yield lower mAP scores than the robots: even though they detect more people overall, the clusters they form are less consistent. Two additional factors influence performance. First, the number of people to be detected has a direct impact: as the number increases, the likelihood of errors rises, leading to lower overall performance. Second, the number of robots or cameras also plays a role, albeit more subtly. A higher number of robots leads to more frequent interaction between them. Without communication, this is only detrimental, as robots waste time avoiding one another and may occlude each other’s field of vision. For CCTV cameras, lower performance with more cameras may also result from the presence of robots: since cameras sometimes misidentify robots as people, and a higher robot count increases misclassification errors. However, when communication is enabled, a greater number of robots or cameras results in more acquired data. As a consequence, the CMC curve and purity improve, while mAP stagnates or slightly declines due to the accumulation of errors. This suggests a trade-off between the amount of shared data and the consistency of the clusters formed by the agents. Overall, the results indicate that communication substantially enhances the Re-ID capability of both robots and cameras.

We then examine the impact of communication frequency on the robots’ performance (see Figure 3). Increasing the communication range—and consequently the communication frequency—generally improves performance, as robots can share more data. However, the results confirm the previously identified trade-off: in experiments with 8 robots, while the CMC curve improves, mAP initially decreases before increasing, and purity eventually decreases. Since a larger number of robots leads to more frequent encounters, there is a threshold where additional meetings enhance the detection of new people but simultaneously reduce cluster consistency as more misidentifications may happen. Depending on the application and the number of available robots, the communication range should be carefully adjusted to optimize performance.

**FIGURE 4:**
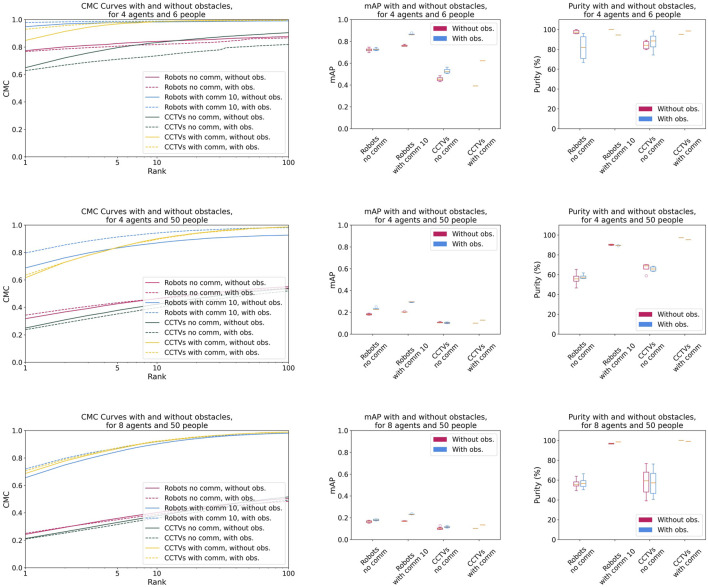
CMC, mAP, and purity with and without obstacles for 4 agents (robots or CCTV cameras) and 6 people, 4 agents and 50 people, and 8 agents and 50 people.

Finally, we examine the impact on performance of obstacles obstructing vision (see Figure 4).

We initially expected obstacles to degrade performance across most configurations, with robots being less affected. However, the results show minimal impact, and in some cases, obstacles even yield positive effects. Specifically, obstacles tend to slightly improve CMC and mAP performance while reducing purity. We hypothesize that while obstacles introduce occlusions, they also increase the separation between people in the environment. As a result, agents observe fewer people at a same time, simplifying detection. This finding may also indicate that the Re-ID method is robust to occlusions, enabling agents to correctly re-identify people even after they disappear behind an obstacle. A thorough investigation would be needed to explore this further.

## 6 Conclusions

The results show that a decentralized approach leveraging swarm perception holds significant potential for person re-identification. By exchanging data in a decentralized manner, robots detect more people and refine their knowledge of those they have already seen. Moreover, mobile agents offer inherent advantages for Re-ID, as even robots without communication outperform fixed CCTV cameras. In a controlled, enclosed environment, a centralized system with optimally placed cameras and continuous communication still achieves slightly better results in most cases. However, its database may be less consistent than that created by the robots, and such a system is not always feasible, particularly in open environments where uncontrollable factors, such as changing lighting conditions or moving obstacles, can disrupt its functionality.

Despite these advantages, challenges remain, particularly when multiple people exhibit high visual similarity. Additionally, the limited field of view caused by the robots’ low height may hinder their performance in densely crowded environments.

Future work will focus on three key directions: enhancing the re-identification pipeline, improving communication and data sharing strategies, and extending the system to more realistic deployment scenarios. First, we plan to augment the re-identification process by incorporating additional modalities—such as spatial and temporal cues within embeddings—and exploring complementary techniques like face recognition. While our current pipeline uses standard components, integrating more discriminative models and advanced merging strategies (e.g., those from the CARPE-ID framework (Rollo et al., 2024)) could reduce clustering errors and improve performance in more challenging conditions. Second, we will investigate selective and adaptive data-sharing mechanisms. Rather than sharing all available data, robots could exchange compressed representations, such as statistical summaries or representative embeddings, to reduce communication load and increase scalability. We also aim to explore federated learning-inspired approaches, where robots share model updates instead of raw data, enabling collaborative adaptation without centralized coordination. Finally, we intend to expand our simulator and testbed to include dynamic and unstructured environments, such as outdoor public spaces or emergency scenarios, where decentralized swarm perception is especially relevant. This includes exploring more sophisticated exploration behaviors that allow robots to actively adapt coverage and reallocate resources based on perceptual uncertainty or mission goals. Moreover, experiments with physical robots would provide critical insight into the system’s real-world transfer, where uncontrolled factors (e.g., lighting variation, dynamic obstacles) and physical limitations (e.g., sensor noise, communication loss, and latency) may degrade performance.

## Data Availability

The raw data supporting the conclusions of this article will be made available by the authors, without undue reservation.
